# A case report of low grade fetal lung adenocarcinoma with TP53 mutation

**DOI:** 10.1097/MD.0000000000029047

**Published:** 2022-03-18

**Authors:** Bo Wang, Huri Jin

**Affiliations:** ^a^ *Department of Surgery, Yanbian University, Yanji, Jilin, China,* ^b^ *Department of Thoracic Surgery Medicine, Affiliated Hospital of Yanbian University, Yanji, Jilin, China.*

**Keywords:** adenocarcinoma fetal, low grade, lung neoplasm

## Abstract

**Rationale:**

Fetal lung adenocarcinoma (FLAC) is a rare malignant tumor that occurs in the alveolar epithelium. FLAC, as a distinct entity, is a malignancy with a very low incidence, accounting for less than 0.5% of all lung tumors, with a high rate of misdiagnosis due to its rarity, lack of typical presentation and imaging signs. According to histopathological differences, FLAC is further divided into 2 types: low-grade FLAC and high-grade FLAC. In the article, we report a young woman who was diagnosed with low-grade fetal-type lung adenocarcinoma.

**Patient concerns:**

An 18-year-old female patient was admitted due to cough and chest distress.

**Diagnosis:**

The final pathological examination confirmed that the lesion was a low-grade fetal lung adenocarcinoma.

**Interventions:**

The patient underwent thoracoscopic left lower lobectomy and regional lymph node dissection.

**Outcomes:**

The postoperative course was stable, and no recurrence was observed 1 year after operation.

**Lessons:**

To the best of our knowledge, there are no previous case reports of low-grade fetal-type adenocarcinoma, TP53 gene mutation, and the significance of its mutation is not extensively studies. FLAC, although extremely rare, is considered in the differential diagnosis of lung cancer. In addition, biopsy, histopathology, and specific immunohistochemical staining of larger tissue specimens are helpful for accurate diagnosis of FLAC.

## 1. Introduction

Fetal lung adenocarcinoma is a rare malignancy arising in the alveolar epithelium and is a variant of lung adenocarcinoma with a histological morphology resembling that of the developing fetal lung. It was first described by Barnard in 1952 as a biphasic tumor of the lung, surrounded by an interstitial matrix similar to the fetal lung, and was therefore named “pulmonary embryonal tumor”.^[1]^ In 1961, Spencer introduced the term pulmonary blastoma (PB) after studying 3 similar cases.^[2]^ In 1982, classified pneumoblastoma composed only of malignant primitive epithelial components similar to fetal adenocarcinoma as “well-differentiated fetal adenocarcinoma (WDFA)”, also known as “epithelial pneumoblastoma”.^[3]^ In 1999, the World Health Organization (WHO) removed WDFA from the PB category and classified it as a variant of lung adenocarcinoma.^[4]^ In 2011, the International Multidisciplinary Classification of Lung Adenocarcinoma established by the International Association for the Study of Lung Cancer (IALSC), the American Thoracic Society (ATS), and the European Respiratory Society (ERS) classified fetal lung adenocarcinoma (FLAC) as a rare variant of invasive lung adenocarcinoma, and classified FLAC into 2 types: low-grade fetal lung adenocarcinoma (L-FLAC) and high-grade fetal lung adenocarcinoma (H-FLAC).^[5]^ In China, the prevalence of L-FLAC and H-FLAC in Chinese patients with primary lung adenocarcinoma has been reported in the literature as 0.32% and 0.54%, respectively.^[6]^ L-FLAC usually appears in the early stages and it has a lower mortality rate compared to H-FLAC. L-FLAC and H-FLAC are generally well-defined solitary masses, and their extent of disease is often confined to 1 site, relying on the peripheral zone, and early patients generally have no obvious clinical manifestations. Therefore, most cases are diagnosed by incidental findings on chest radiography. Some patients may present with corresponding compression symptoms as the tumor continues to enlarge.^[7-9]^ Surgical treatment to remove the tumor is currently the primary treatment and careful postoperative follow-up. The differential diagnosis of FLAC includes pneumoblastoma, adenoalveolar adenocarcinoma, carcinoid, adenosquamous carcinoma, neuroendocrine carcinoma, and metastatic endometrial adenocarcinoma of the lung. The diagnosis of FLAC should be combined with imaging findings, histopathological examination, and immunohistochemical correlative examination. In this paper, we report a rare case of low-grade fetal lung adenocarcinoma with no specific clinical manifestations and challenging to diagnose by imaging examination. In this case, histopathological examination was performed by video-assisted thoracoscopic surgery (VATS) to confirm the diagnosis.

## 2. Case report

An 18-year-old female was admitted due to “cough and chest distress for 2 years” on November 30, 2020. The patient had occasional dry cough and chest distress after activities 2 years before admission, but did not pay attention to it. On November 21, 2020, the patient visited a local hospital for physical examination and was found to have a mass in the left lower lobe. Enhanced chest computed tomography (CT) showed a mass lesion in the left lower lobe, which tended to have a benign interstitial origin, with a greater possibility of chondroma. For further diagnosis and treatment, the patient visited our outpatient department, and was admitted due to space-occupying lesions in the lung. The patient was healthy in the past, without history of hypertension, diabetes, heart disease, hepatitis, tuberculosis or other medical history or relevant contact history; the patient denied any history of surgery, trauma, blood transfusion, allergy or specific vaccination. Family history revealed healthy parents, no siblings, and no relevant medical history.

Physical examination findings revealed a body temperature of 36.6°C; Pulse - 80bpm; Respiratory rate - 18times/min; Blood pressure - 120/76 mm Hg; clear mind, normal development, general nutrition status, normal mental status, normal gait. The patient had symmetrical thorax, no deformity, equal respiratory activity; no tremor, no obvious pleural friction sensation, unvoiced percussion; slightly weak breath sounds in the left lung, no obvious dry or moist rales heard.

Laboratory test results: 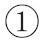 Blood routine and various biochemical tests showed: platelet:465 × 10^9^/L; eosinophil percentage: 5.7%; platelet volume: 0.47%; creatine phosphokinase: 804IU/L; lactate dehydrogenase: 231IU/L; creatine phosphokinase isoenzyme: 26IU/L; the above 6 items were increased to varying degrees, and the others were normal. The results of coagulation function, liver and kidney function, urine routine, infection showed no significant abnormality; 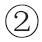 arterial blood gas was normal; 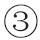 blood test showed normal serum calcium, serum phosphorus, serum potassium, serum sodium, and serum chloride; 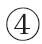 tumor markers showed no significant abnormality. Imaging examination: 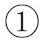 Electrocardiogram, echocardiography, hepatobiliary, pancreatic, splenic and urinary color ultrasound, carotid artery and vertebral artery color ultrasound was normal. 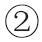 Contrast-enhanced CT of the chest revealed a mass lesion in the left lower lobe, which tended to have a benign stromal origin and was more likely to be chondroma (Fig. 1A and B). 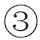 The results of pulmonary function test were normal, without obvious shortness of breath or dyspnea. The breath holding test was up to 35 second, and the stair climbing test was on the 7th floor.

**Figure:**
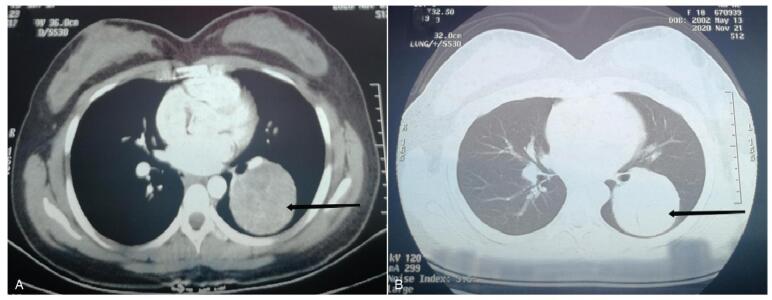
**Figure 1.** Technique: Chest enhanced CT. (A) and (B) showed a mass in the left lower lobe, about 6 cm in diameter, with clear boundary and less uniform density. CT=computed tomography.

The diagnosis was left lower lobe mass of unknown nature. On December 3, 2020, thoracoscopic left lower lobectomy and regional lymph node dissection were performed under general anesthesia. Intraoperatively, the tumor was located in the left lower lobe, measuring about 5.8 cm × 5.8 cm × 5.0 cm, hard in consistency, well-defined, and multiple enlarged lymph nodes at the hilum. Pathological finding was suggestive of low-grade fetal adenocarcinoma (left lower lobe) (Fig. 2A and B), showing infiltrative growth, with massive necrosis, no accumulation of capsule and bronchial sialorrhea tissue, no tumor cells were seen at the bronchial stump. No cancer metastasis was observed in the submitted lymph nodes (0/2): parabronchial (0/1), 5 groups (0/1). Immunohistochemical results showed that the tumor cells showed TTF-1 (+), CK (+), CK7 (focal +), CgA + (small part +), NapsinA (small part +), Syn (small part +), CEA (-), and Ki- (60% + at some parts). Genetic test results showed that a total of 7 mutations in 5 genes were detected, TP53 (exon8: c.868C>T: p.R290C), ARID1A (exon12: c.3386A>G: p.K1129R, exon13: c.3521C>T: p.P1174L), CTNB1 (exon4: c.80G>T: p.G27V), JAK1 (exon10: c.1390G>A: p.V464M) MSH6 (exon3: c.1175G>A: p.C392Y, exon3: c.817G>A: p.D273N). The patient recovered well after surgery without complications, and regular follow-up did not show recurrence to date.

**Figure:**
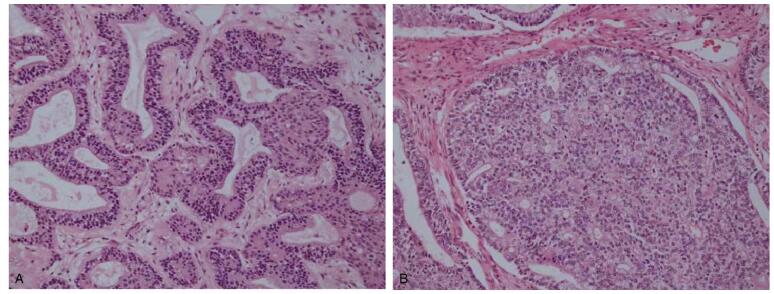
**Figure 2.** Technique: Low power field (A, original magnification ×100) microscopic images with hematoxylin and eosin stains from the tumor. Findings: (A) shows that the tumor is mainly composed of glandular and tubular structures, and the tumor cells are arranged columnar, with small size and relatively uniform nuclei; (B) shows that the local glandular structure is complex, vacuoles are observed on or under the nucleus, and mulberry bodies are observed in some glandular cavities.

## 3. Discussion

FLAC is a rare malignant lung tumor. It accounts for 0.1% to 0.5% of all lung tumors.^[7,10,11]^ It was first reported in 1982 by and described as a subtype of pneumoblastoma.^[12]^ In 1984, Kodama et al introduced the word “fetal-type lung adenocarcinoma” to further classify these tumors.^[13]^ In 1999, the tumor was classified by the World Health Organization (WHO) as a variant of lung adenocarcinoma and is currently called “fetal lung adenocarcinoma” (FLAC). In 2011, the new international multidisciplinary classification of lung adenocarcinoma first divided it into 2 types: L-FLAC and H-FLAC.^[5]^

FLAC’s mass is generally solitary, well-circumscribed, and located in the periphery of the lung. About 25% to 40% of patients are asymptomatic at presentation, most of which are incidentally found by chest radiography,^[2,7]^ and usually with the increasing size of the tumor, corresponding compression symptoms may occur, which may present with chest pain, chest tightness, dyspnea, and cough and hemoptysis. However, L-FLAC and H-FLAC also exhibit different clinical features. H-FLAC is more common in elderly male patients around 60 to 70 years of age, mostly with a history of smoking. The age of onset of L-FLAC then tends to be mostly in young women aged 30 to 40 years. Patients with L-FLAC usually present with stage I-II disease, whereas patients with H-FLAC usually present with more advanced disease (stages III-IV).^[5]^

FLAC is an adenocarcinoma resembling the developing fetal lung in the pseudoglandular stage (8-16weeks of gestation). It is composed of complex, branched tubular glands lined by glycogen-rich, non-ciliated columnar or cuboidal cells. Cells have clear cytoplasm, large vesicular nuclei, and supra- or subnuclear vacuoles that are morphologically similar to early secretory endometrium.^[6,14]^ Solid cell spheres formed by scalelike cells, the characteristic “mulberry bodies”, are often seen at the base or in the glandular duct.^[15]^ L-FLAC showed a purely histological pattern with distinct “mulberry body” structure formation and low nuclear atypia. However, H-FLAC usually shows more than 50% fetal morphology, as well as coexistence with other common types of lung adenocarcinoma. Moreover, H-FLAC usually exhibits marked nuclear atypia and nucleoli with necrosis and high frequency of mitoses without “mulberry body” structure formation.^[8,16]^

These 2 types of FLAC also differ in terms of immunohistochemical features. Both L-FLAC and H-FLAC showed positivity for neuroendocrine markers (Cg-A, Syn, TTF-1). In this patient, the test results of Cg-A, Syn, and TTF-1 were positive. It was initially thought that the positive ratio of neuroendocrine markers was more pronounced in L-FLAC than in H-FLAC. But recent studies have shown that its positive ratio in H-FLAC depends on the percentage of fetal lung-like component present in the tumor.^[8,17]^ L-FLAC constantly showed abnormal β-catenin nuclear/cytoplasmic expression and frequent β-catenin gene mutations. In contrast, H-FLAC shows membranous staining for β-catenin, while lacking β-catenin gene mutations.^[18-20]^

A large number of molecular studies have been statistically analyzed, and the mutation rates of KRAS, EGFR, and PIK3CA in FLAC are very low. Only a few cases in China have found L858R point mutation of EGFR in H-FLAC and T790MEGFR mutation in L-FLAC.^[6]^ In this case, KRAS, EGFR, and PIK3CA mutations occurred. TP53 encodes the p53 tumor suppressor protein, a transcription factor that responds to cellular stress (including DNA damage and oncogenic activation) by inducing downstream antitumor responses such as DNA repair and apoptosis. Mutations in TP53 also differ greatly in L-FLAC and H-FLAC, which show nuclear p53 overexpression, but p53 expression is found in L-FLAC.^[6,8]^ Bodner and Koss found through a large case collection that no TP53 mutation was contained in L-FLAC cases, that mutations in TP53 were present in H-FLAC, and that their mutation frequency was similar to that of conventional lung cancer. This may illustrate the lack of TP53 mutations in L-FLAC cases indicating that the good prognosis of this tumor may be related to fewer molecular alterations.^[21]^ However, in this case, TP53 was mutated (exon8: c.868C>T: p. R290C), and its mutation abundance was 1.8%, which is different from the reported related cases, and more cases need to be accumulated and studied on the role of TP53 mutation in the development of L-FLAC.

The frequency of CTNNB1 mutations in L-FLAC is high, and some scholars in China believe that CTNNB1 mutations are typical genetic changes in L-FLAC. This is because, CTNNB1 exon 3 mutations are associated with reduced β-catenin degradation, β-catenin accumulation in the cytoplasm, and subsequent translocation to the nucleus, which reasonably explains the immunophenotypic characteristics of β-catenin in L-FLAC.^[22]^ This patient also had a mutation in CTNNB1 (exon4: c.80G > T: p.G27V).

FLAC should be differentiated from the following diseases: 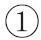 Pulmonary blastoma: Pulmonary blastoma is composed of primitive epithelial and mesenchymal components, and the epithelial component tumor cells are monolayer cuboidal, arranged in glandular tubes, scattered in immature mesenchymal tissues, while FLAC stroma is benign and can be differentiated from it. 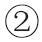 Vesicular adenocarcinoma: Endometrioid glands characteristic of FLAC are not pathologically present, and fibrotic stroma is common, while FLAC stroma is highly vascular. 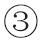 Carcinoid tumor: neuroendocrine granules were shown under ultrastructure, and Syn, Cg-A, and CD56 showed diffuse positivity in immunohistochemistry. 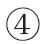 Adenosquamous carcinoma: Adenosquamous carcinoma includes adenocarcinoma component and squamous cell carcinoma component, of which keratinized beads and intercellular bridges are seen in scaly cell carcinoma, but only adenocarcinoma component is present in FLAC, which can be differentiated. Second, scale-cell carcinoma CK5/6, p63, and TTF-1 were not expressed. 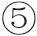 Neuroendocrine carcinoma: the tumor cells are arranged in a beam-like, solid and glandular pattern, during which the blood vessels in the cytoplasm are abundant, and the neuroendocrine markers are expressed in the tumor cells.6 Metastatic endometrial adenocarcinoma of the lung: Female patients are usually gynecological symptoms, and uterine or ovarian masses can be found by examination. It is divided into 2 types: well-differentiated and poorly differentiated. Well-differentiated endometrial adenocarcinoma glands fuse with each other and branch to form labyrinthine structures. When scaly mulberry bodies may occur in some cases, they are histologically similar to L-FLAC at this time, but endometrioid adenocarcinoma often shows necrosis and desmoplastic stroma, which can be differentiated. Poorly differentiated endometrial adenocarcinoma was histologically similar to H-FLAC, but in immunohistochemistry, endometrial adenocarcinoma was positive for ER, PR, and vimentin, but negative for TTF-1 expression.

There is no standard treatment for FLAC internationally, and surgical resection of the lesion is currently the main treatment for FLAC, with radiotherapy and chemotherapy as adjuvant therapy after surgery in some cases, but the therapeutic effects of radiotherapy and chemotherapy are relatively limited.^[23]^ It has also been reported that after preoperative platinum-based chemotherapy for patients, the tumor shrank significantly, facilitating the resection of the tumor.^[24]^

L-FLAC is usually early at onset and rarely develops regional lymph node metastasis and distant metastasis,^[25]^ all of which have a good prognosis with a 10-year survival rate of >75% in patients. However, the clinical stage found by H-FLAC is generally high, and there are often regional lymph node metastasis and distant metastasis on examination, so its prognosis is poor, with a 5-year survival rate of only 53.6%.^[8]^ Nakatani et al^[19,20]^ suggested that malignancy grade and tumor stage accounted for the prognostic differences observed in these 2 studies and further noted that although it is unclear whether H-FLAC has a worse prognosis than stage-matched conventional adenocarcinoma, L-FLAC may have a better prognosis than stage-matched conventional lung adenocarcinoma.

## Author contributions

**Conceptualization:** Bo Wang.

**Investigation:** Bo Wang.

**Project administration:** Huri Jin.

**Supervision:** Huri Jin.

**Writing** - **original draft:** Bo Wang.

**Writing** - **review & editing:** Bo Wang, Huri Jin.
